# Development and evaluation of a method to assess breast cancer risk using a longitudinal history of mammographic density: a cohort study

**DOI:** 10.1186/s13058-023-01744-y

**Published:** 2023-11-24

**Authors:** Emma C. Atakpa, Diana S. M. Buist, Erin J. Aiello Bowles, Jack Cuzick, Adam R. Brentnall

**Affiliations:** 1 Wolfson Institute of Population Health, Barts and The London School of Medicine and Dentistry, Queen Mary University of London, London, EC1M 6BQ UK; 2 Kaiser Permanente Washington Health Research Institute, Seattle, WA USA; 3 Kaiser Permanente Bernard J Tyson School of Medicine, Pasadena, CA USA

**Keywords:** Mammographic density, Longitudinal data, Repeated measures, Breast cancer risk, Mammography screening

## Abstract

**Background:**

Women with dense breasts have an increased risk of breast cancer. However, breast density is measured with variability, which may reduce the reliability and accuracy of its association with breast cancer risk. This is particularly relevant when visually assessing breast density due to variation in inter- and intra-reader assessments. To address this issue, we developed a longitudinal breast density measure which uses an individual woman’s entire history of mammographic density, and we evaluated its association with breast cancer risk as well as its predictive ability.

**Methods:**

In total, 132,439 women, aged 40–73 yr, who were enrolled in Kaiser Permanente Washington and had multiple screening mammograms taken between 1996 and 2013 were followed up for invasive breast cancer through 2014. Breast Imaging Reporting and Data System (BI-RADS) density was assessed at each screen. Continuous and derived categorical longitudinal density measures were developed using a linear mixed model that allowed for longitudinal density to be updated at each screen. Predictive ability was assessed using (1) age and body mass index-adjusted hazard ratios (HR) for breast density (time-varying covariate), (2) likelihood-ratio statistics (ΔLR-*χ*^2^) and (3) concordance indices.

**Results:**

In total, 2704 invasive breast cancers were diagnosed during follow-up (median = 5.2 yr; median mammograms per woman = 3). When compared with an age- and body mass index-only model, the gain in statistical information provided by the continuous longitudinal density measure was 23% greater than that provided by BI-RADS density (follow-up after baseline mammogram: ΔLR-*χ*^2^ = 379.6 (degrees of freedom (*df*) = 2) vs. 307.7 (*df* = 3)), which increased to 35% (ΔLR-*χ*^2^ = 251.2 vs. 186.7) for follow-up after three mammograms (*n* = 76,313, 2169 cancers). There was a sixfold difference in observed risk between densest and fattiest eight-category longitudinal density (HR = 6.3, 95% CI 4.7–8.7), versus a fourfold difference with BI-RADS density (HR = 4.3, 95% CI 3.4–5.5). Discriminatory accuracy was marginally greater for longitudinal versus BI-RADS density (c-index = 0.64 vs. 0.63, mean difference = 0.008, 95% CI 0.003–0.012).

**Conclusions:**

Estimating mammographic density using a woman’s history of breast density is likely to be more reliable than using the most recent observation only, which may lead to more reliable and accurate estimates of individual breast cancer risk. Longitudinal breast density has the potential to improve personal breast cancer risk estimation in women attending mammography screening.

**Supplementary Information:**

The online version contains supplementary material available at 10.1186/s13058-023-01744-y.

## Background

Mammographic density is one of the strongest known risk factors for breast cancer [1]. Mammographic density is commonly measured using the Breast Imaging Reporting and Data System (BI-RADS) four-category scale, summarised as (A) almost entirely fat, (B) scattered fibroglandular, (C) heterogeneously dense or (D) extremely dense breasts [2]. Including BI-RADS density in breast cancer risk models improves the accuracy of individual risk assessment [3]. Providing accurate estimation of a woman’s breast cancer risk could aid decisions regarding supplemental screening, risk-reducing strategies based on diet and exercise, preventive therapy and risk-stratified screening.

Most studies assessing the effect of mammographic density on breast cancer risk are based on a single measurement. Whilst some studies measure density at more than one time point, the majority of studies assess change in density between mammograms taken at two time points [4, 5]. Moreover, the focus of many of these studies is on the association between density change and breast cancer risk, rather than the predictive ability of multiple serial measurements of density. A recent systematic review [4] identified just one study that had evaluated the predictive performance of using more than one serial density measurement for risk of subsequent breast cancer [6]. This US cohort study of ~ 700,000 women explored using two density values over two years to assess invasive breast cancer risk and observed a small improvement in risk discrimination (concordance index 0.640 vs. 0.635) [6]. However, using a woman’s entire history of breast density measurements might be more informative for breast cancer risk estimation than using only one or two time points. This is partly because mammographic density is a dynamic trait that decreases most strongly with increasing body mass index (BMI) [7, 8], age [9, 10] and during the menopause [10, 11]. It also decreases in response to endocrine treatment [12–15] and increases with current hormone replacement therapy use [16–18]. There is also variation in radiologists’ visual interpretation of mammographic density due to inter- and intra-reader variability [19], and thus, there is a need for measurements of breast density to be more reliable than they are at present [20]. Using a woman’s history of previous density measurements should help to reduce such measurement error. In addition, when using a breast density history with more than two records, it may be important to include information on mammograms arbitrarily spaced through time, i.e. not necessarily only two mammograms approximately one or two years apart, but previous work does not appear to have considered this.

## Methods

The aim of this cohort study was to develop a method to assess breast cancer risk using a longitudinal history of breast density and to assess the predictive value of this approach in comparison with breast density measured at a single time point.

### Setting and study population

This analysis included women from the Kaiser Permanente Washington Breast Cancer Surveillance Consortium (BCSC) breast imaging registry (National Cancer Institute RRID:SCR_011403) [21, 22], an integrated healthcare system that provides insurance and health care in Washington State. The cohort has previously been used to assess the long-term performance of breast cancer risk assessment with and without density [3]. Women in the cohort attended screening from 1 January 1996 to 31 December 2013 (with follow-up from 1 January 1996 to 31 December 2014) with no prior diagnosis of invasive breast cancer, ductal carcinoma in situ (DCIS) or lobular carcinoma in situ at study entry. Exclusions were made for women aged < 40 yr or > 73 yr at their baseline screening mammogram and women with less than 6-month follow-up (to obtain a cohort who were breast cancer-free within 6 months of their baseline screening mammogram).

### Endpoints

The primary outcome was diagnosis of invasive breast cancer. Women were followed up from 6 months after their first screening mammogram with an available density assessment (baseline mammogram) until the earliest of: diagnosis of invasive breast cancer or censoring (age 75 yr, the recommended end of screening age; 31 December 2014, the end of calendar time follow-up; DCIS; death; or health plan disenrollment). Outcomes were obtained through electronic health data and linkage with the regional population-based Surveillance, Epidemiology, and End Results tumour registry and pathology databases.

### Exposure variables

Mammographic density was recorded by the interpreting radiologist using BI-RADS density categories (A = almost entirely fat, B = scattered fibroglandular, C = heterogeneously dense or D = extremely dense). Mammograms missing BI-RADS density were excluded (24,707/721,406; 3%). Self-reported height and weight were collected using a questionnaire completed at each screening mammogram to calculate BMI (dividing weight (kg) by height (m) squared). Self-reported race and ethnicity were collected at baseline questionnaire. Values were checked for validity at the time of scanning for research purposes. A flow chart detailing availability of mammograms and women included in the study is shown in Additional file 1: Fig. S1.

### Longitudinal breast density measures

Continuous longitudinal density measures were derived from a linear mixed model with BI-RADS density as the outcome variable (treated as an integer 1–4). The model form was determined based on iterative testing and examination of model fit. The final model was fitted by maximum likelihood with fixed effects for intercept, age (per 5 yr; quartic polynomial terms; continuous), BMI (per kg/m^2^; continuous) and interaction between age (per 5 yr; linear term only) and BMI. Intercept and time (age) were treated as random effects with an unstructured 2 × 2 covariance matrix. The continuous longitudinal density measure at each time point was the predicted density from the model using the data observed to that point, following an empirical Bayes approach [23]. Briefly, empirical Bayes is a statistical method which estimates a prior probability distribution using the observed data. Standard errors for the model parameters were based on robust sandwich estimators [24–26]. The main measure of interest is continuous longitudinal breast density. However, in order to help with clinical interpretations of the hazard ratios (HRs) associated with the continuous longitudinal density measure, we also derived a categorical variable for longitudinal breast density (i.e. to show the spread of risk when using smaller bins, which will eventually approach a continuous measure). To do this, continuous longitudinal density was categorised with cut points chosen so that the percentage of women at baseline in categories 1–2 matched BI-RADS A, categories 3–4 matched BI-RADS B, etc.; and there was an equal split within categories 1–2, 3–4, 5–6 and 7–8, e.g. the lower 50% of BI-RADS A at baseline was in category 1 and the upper 50% was in category 2. An intuitive explanation of the longitudinal breast density measure is described in Additional file 1: Methods.

### Statistical analysis

BMI was missing for 5% of baseline mammograms (6047/132,439) and 16% of follow-up mammograms (72,539/450,189). Missing BMI at baseline was imputed using the sample mean BMI given the woman’s age at baseline. Missing BMI at follow-up mammograms was carried forward from the woman’s last recorded BMI. Therefore, all women had density, age and BMI at each mammogram used in the final dataset. BMI was winsorised for values below 15 kg/m^2^ [baseline: 155/132,439 (0.1%); follow-up: 351/450,189 (0.1%)] and above 35 kg/m^2^ [baseline: 18,410/132,439 (14%); follow-up: 66,418/450,189 (15%)]. This was done so that women who were morbidly obese would have the same breast cancer risk due to adiposity as women who were obese.

Proportional-hazards models were fitted with one of the three time-varying density covariates measured at each mammogram: (1) BI-RADS categorical density; (2) continuous longitudinal density; and (3) eight-category longitudinal density. All models were adjusted for age at baseline (per yr; continuous) and time-varying BMI (per kg/m^2^; continuous). All time-varying variables were updated at each screening examination. To allow for a possible nonlinear relationship, density was treated as a factor variable for models 1 and 3 (degrees of freedom (*df*) = 3 and 7, respectively), and model 2 included linear and quadratic terms (*df* = 2) related to the continuous longitudinal density measure. Age was sometimes presented as categories for younger or older than 55 yr as a crude approximation for pre- or postmenopausal, respectively. Likelihood-ratio statistics (ΔLR-*χ*^2^(*df*)) were from the proportional-hazards models, relative to a proportional-hazards model including age and BMI only. A concordance index (yC) was estimated to measure discriminatory ability (Additional file 1: Methods), with 95% confidence intervals (95% CI) from empirical bootstraps with 10,000 resamples.

Primary analysis tested the continuous longitudinal density measure on all women in the cohort, starting from their first mammogram. Since the benefit was expected to be greater in women with more than one mammogram, a secondary analysis started from women’s third mammogram, in those with ≥ 3 mammograms.

To test robustness, we did sensitivity analyses using the complete cohort but excluding mammograms < 6 months before cancer diagnosis (incident screen-detected mammograms) and excluding mammograms with imputed BMI, as well as evaluating the predictive performance of longitudinal density compared with BI-RADS density at baseline, and when it is defined as a four-category variable (e.g. categories 1–2 for eight-category longitudinal density equal category 1 as a four-category variable). The evaluation of longitudinal density against baseline BI-RADS density was conducted because, according to a model by Boyd et al. [27, 28], rates of breast tissue ageing are higher at younger ages due to higher levels of hormone exposure; therefore, baseline mammograms (representing breast density at a younger age than time-varying mammograms) may be more reflective of a woman’s ‘peak’ available breast density and hence be more informative for risk prediction.

We hypothesised that longitudinal density would be more stable through time than BI-RADS density. To test this, we plotted cumulative distribution functions for relative risk of BI-RADS density or longitudinal density from second to third mammogram (hazard ratios from the proportional-hazards Cox model fitted to the complete cohort, including age at baseline and BMI), in women with ≥ 3 mammograms.

Exploratory analyses considered potential improvements to the proportional-hazards model, including use of trajectories (random effect slopes) to reflect likely future within-woman rate of change, and interactions between density and age, BMI or race. The primary proportional-hazards model was also stratified by age at baseline (< 55 yr representing premenopausal and ≥ 55 yr representing postmenopausal) to assess whether the performance of the longitudinal measure differed by menopausal status. Two exploratory proportional-hazards models were also fitted with oestrogen receptor positive or negative subtype as the outcome (with censoring of the complementary subtype). Heterogeneity by oestrogen receptor subtype was evaluated using a case-only logistic regression model with subtype as the outcome, covariates for age, BMI and continuous longitudinal breast density (linear and quadratic; calculated using mammograms up to and including the screening visit immediately before the date of diagnosis or censoring, with corresponding age and BMI also from the immediately preceding screening visit), and associated p-values for longitudinal breast density from Wald tests. We also evaluated longer-term risk of longitudinal density using breast density at (BI-RADS)/to (longitudinal) the third mammogram only and not updating it further through follow-up time.

Analysis was conducted using Stata version 13 (RRID:SCR_012763) [29] and R version 3.3.3 (RRID:SCR_000432) [30], with two-sided hypothesis tests.

## Results

The cohort included 132,439 women with a median follow-up of 5.2 yr (interquartile range (IQR) 2.4–11.1 yr) and maximum follow-up of 19 yr. The median time between mammograms was 1.8 yr (IQR 1.0–2.0 yr) and the median number of mammograms per woman was 3 (IQR 2–6), with 32,010 women (24.2%) having a baseline mammogram only. The number of mammograms was similar across different ages at baseline and throughout the follow-up. Women were of Asian (9%), Black (4%), Mixed (3%), Other (3%), Unknown (2%) or White (80%) race, and Hispanic ethnicity was reported in 5% of women. In total, 2704 women (2.0%) were diagnosed with invasive breast cancer during follow-up. Summary statistics on age, BMI and breast density at baseline are shown in Table 1.

**Table 1:** Hazard ratios for invasive breast cancer by age, body mass index and BI-RADS density (baseline)

Baseline variable	No. of women (%)	Follow-up, 1000 women-years	No. of invasive breast cancers	Hazard ratio* (95% CI)
Total	132,439 (100)	874	2704	-
Age (years)				
40–49	60,325 (45.6)	418	977	1 [Reference]
50–59	43,878 (33.1)	317	1057	1.41 (1.29–1.53)
60–73	28,236 (21.3)	139	670	2.26 (2.04–2.50)
Continuous	Median (IQR) = 50 (44–58)		LR-*χ*^2^(*df* = 1) = 309.1 (*p* < 0.001)	
BMI (kg/m^2^): < 55 years				
Underweight (< 18.5)	1087 (1.3)	7	13	0.70 (0.41–1.22)
Healthy (≥ 18.5 to 25)	33,808 (38.8)	246	623	1 [Reference]
Overweight (≥ 25 to 30)	26,428 (30.3)	186	513	1.10 (0.98–1.23)
Obese (≥ 30 to 35)	13,126 (15.1)	92	221	0.96 (0.82–1.11)
Morbidly obese (≥ 35)	12,707 (14.6)	87	211	0.98 (0.84–1.15)
Continuous	Median (IQR) = 26.6 (23.0–31.2)		LR-*χ*^2^(*df* = 1) = 0.02 (*p* = 0.89)	
BMI (kg/m^2^): ≥ 55 years				
Underweight (< 18.5)	570 (1.3)	3	12	1.18 (0.66–2.10)
Healthy (≥ 18.5 to 25)	14,905 (32.9)	85	308	1 [Reference]
Overweight (≥ 25 to 30)	16,440 (36.3)	92	449	1.35 (1.17–1.56)
Obese (≥ 30 to 35)	7665 (16.9)	44	194	1.22 (1.02–1.46)
Morbidly obese (≥ 35)	5703 (12.6)	33	160	1.33 (1.10–1.60)
Continuous	Median (IQR) = 27.4 (23.8–31.0)		LR-*χ*^2^(*df* = 1) = 6.9 (*p* = 0.01)	
BI-RADS density				
Fatty	10,387 (7.8)	61	107	0.59 (0.48–0.72)
Scattered	46,206 (34.9)	309	786	1 [Reference]
Heterogeneous	57,158 (43.2)	376	1338	1.76 (1.61–1.93)
Extremely dense	18,688 (14.1)	128	473	2.31 (2.04–2.63)
Continuous	Median (IQR) = 3 (2–3)		LR-*χ*^2^(*df* = 1) = 91.8 (*p* < 0.001)	

*n* = 132,439

95% CIs from Wald tests

LR-*χ*^2^(*df* = 1) is the likelihood-ratio trend test for each variable alone

*BI-RADS*  Breast Imaging Reporting and Data System, *BMI* body mass index, *df* degrees of freedom, *IQR* interquartile range, *95% CI* 95% confidence interval

*Hazard ratios from proportional-hazards Cox models (model for BI-RADS density is adjusted for continuous age and continuous BMI, models for age and BMI are univariable)

**Fig. 1:**
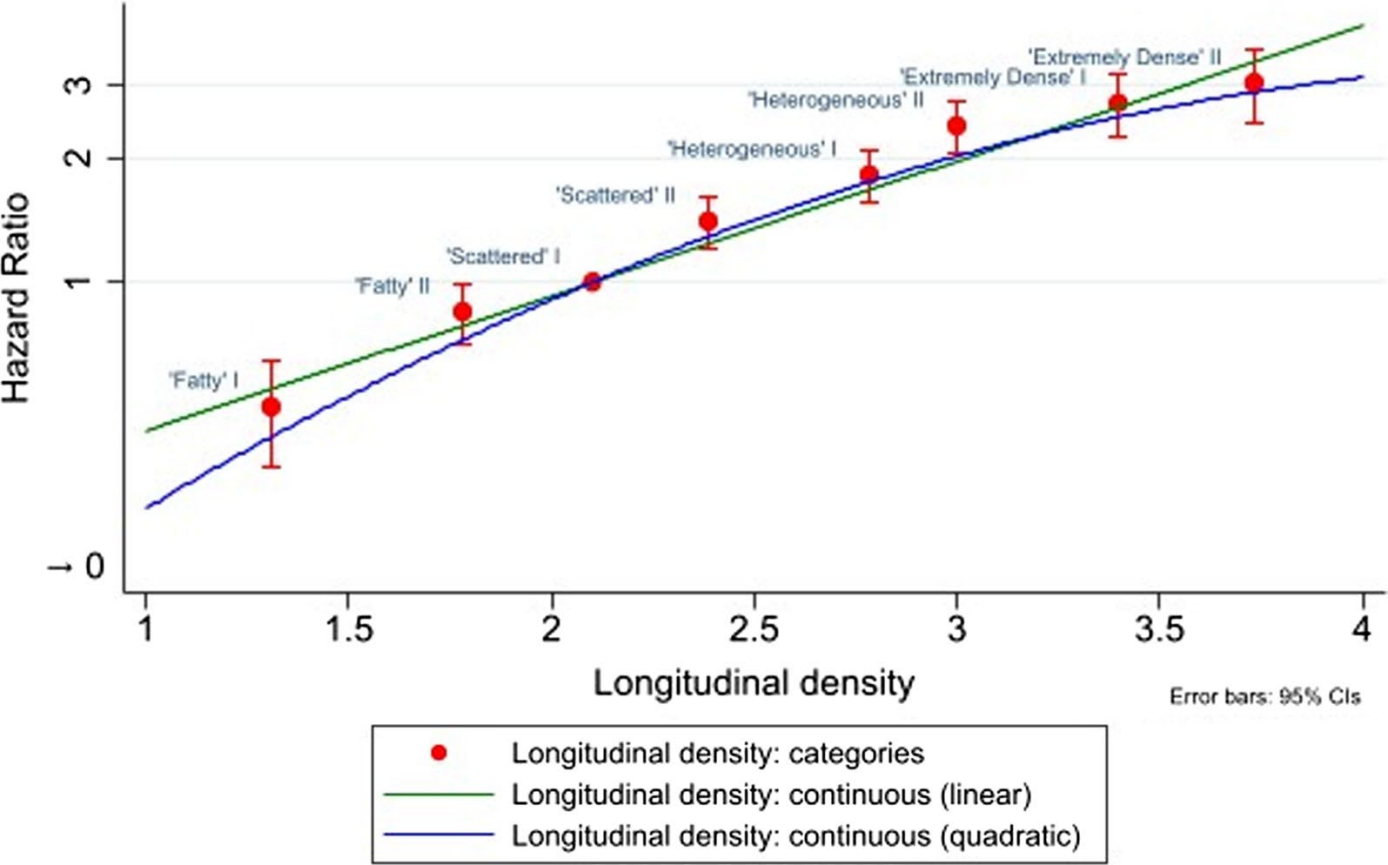
Adjusted hazard ratios for invasive breast cancer by longitudinal breast density (continuous and categorical). *n* = 132,439. Adjusted hazard ratios from proportional-hazards Cox models for the new longitudinal breast density measure (continuous, time-varying) or derived eight-category longitudinal breast density (time-varying) which was based on the continuous longitudinal breast density measure (reference = Scattered I). Hazard ratios adjusted for age at baseline (continuous) and time-varying BMI (continuous). ‘Time-varying’ means that a woman’s values for breast density and BMI are updated through time, i.e. at each screening examination. Categorical longitudinal density on the *x*-axis is the mean of the new longitudinal breast density measure (continuous) in each group. Units of longitudinal density are non-standard. 95% CIs from Wald tests. *Y*-axis on a log scale. Red circles represent the adjusted hazard ratios for invasive breast cancer by categorical longitudinal density, and the error bars represent the 95% CIs for the hazard ratios. Green line represents the adjusted hazard ratios for invasive breast cancer by continuous longitudinal density, fitted with a linear term only. Blue line (representing the final model used in the analysis) represents the adjusted hazard ratios for invasive breast cancer by continuous longitudinal density, fitted with a linear term and a quadratic term. *BMI* body mass index, *95% CI* 95% confidence interval

In the linear mixed model for continuous longitudinal density (Additional file 1: Table S1), an unstructured covariance matrix was chosen because it provided a substantially better model fit than an independent structure (ΔLR-*χ*^2^(1) = 2473.3). A quartic polynomial term for age and an interaction between age and BMI were included because likelihood-ratio statistics and graphical plots of predicted density by age and BMI identified an improved model fit. There was also evidence of a nonlinear relationship between longitudinal density and breast cancer risk (Fig. 1), with improvement in model fit when including a quadratic term (ΔLR-*χ*^2^(1) = 15.0), but not a cubic term (ΔLR-*χ*^2^(1) = 1.1).

**Fig. 2:**
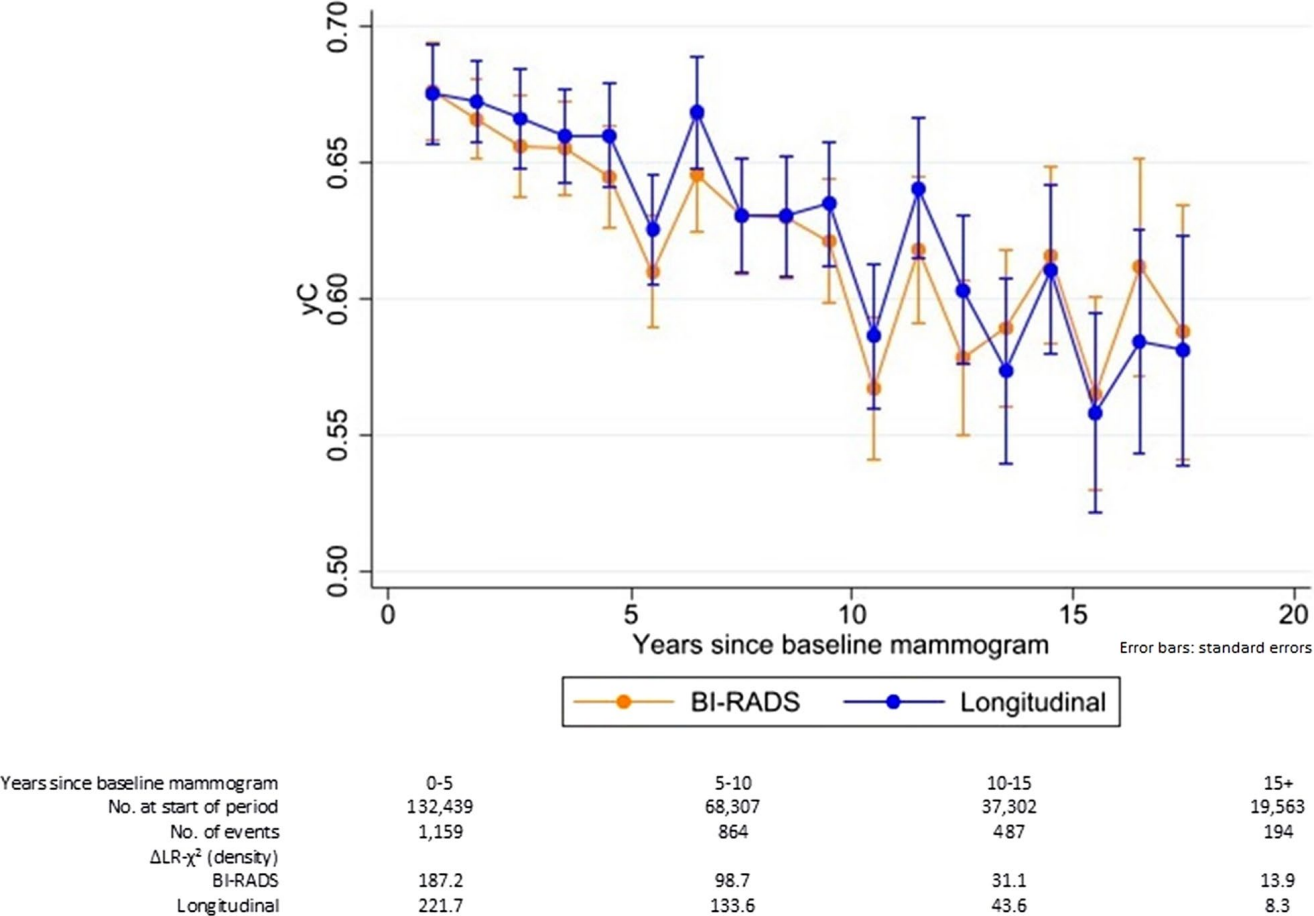
Yearly mean concordance index through time for BI-RADS density or continuous longitudinal breast density. *n* = 132,439. yC is the yearly mean concordance index. ΔLR-*χ*^2^ represents the difference in likelihood-ratio statistics between a model fitted to age at baseline (continuous) and time-varying BMI (continuous) and a model additionally incorporating the density term(s). BI-RADS density and the new longitudinal breast density measure (continuous, including a quadratic term) are time-varying in the proportional-hazards Cox models. ‘Time-varying’ means that a woman’s values for breast density and BMI are updated through time, i.e. at each screening examination. The concordance index decreases through time because the age range in the cohort decreases over time. (Women aged 40 yr get older, and women are censored after age 75 yr.) Orange line represents the yC through time for BI-RADS density. Blue line represents the yC through time for longitudinal density. Error bars represent the standard errors for the yC values at yearly intervals, starting at 0.5 years. *BI-RADS* Breast Imaging Reporting and Data System, *BMI* body mass index

All breast density methods were strongly associated with risk (Table 2), but most statistical information was in the continuous longitudinal density measure. It added 23% more statistical information to an age- and BMI-only model than BI-RADS density (ΔLR-*χ*^2^(2) = 379.6 vs. ΔLR-*χ*^2^(3) = 307.7, respectively). This corresponded to a marginal increase in the mean c-index over all of the follow-up: 0.64 for continuous longitudinal density vs. 0.63 for BI-RADS density (mean difference in c-indices = 0.008, 95% CI 0.003–0.012) (Fig. 2). The eight-category version of longitudinal density was inferior to the continuous measure (ΔLR-*χ*^2^(7) = 375.1), but aids interpretation of the hazard ratios associated with the continuous measure (Table 2). The densest of the eight categories (‘Extremely dense’ II) had threefold greater risk (HR = 3.0, 95% CI 2.5–3.7) than the reference (‘Scattered’ I), with a sixfold greater risk when comparing densest and fattiest breasts (HR = 6.3, 95% CI 4.7–8.7, relative to ‘Fatty’ I). This compared with a fourfold increased risk between densest and fattiest BI-RADS density (Extremely dense HR = 4.3, 95% CI 3.4–5.5, relative to Fatty).

**Table 2:** Adjusted hazard ratios for invasive breast cancer by BI-RADS density or longitudinal breast density (continuous/categorical)

Model	Density measure		Adjusted hazard ratio (95% CI) (time-varying breast density covariate)
1	BI-RADS density		
	Fatty		0.49 (0.40–0.60)
	Scattered		1 [Reference]
	Heterogeneous		1.71 (1.56–1.86)
	Extremely dense		2.11 (1.84–2.42)
			ΔLR-*χ*^2^ (*df* = 3) = 307.7
2	Continuous longitudinal density		
	Linear (per unit*)		5.53 (3.34–9.14)
	Quadratic (per unit^2^*)		0.83 (0.76–0.92)
			ΔLR-*χ*^2^ (*df* = 2) = 379.6
3	Categorical longitudinal density		
	Category 1:	‘Fatty’ I (< 1.5)	0.48 (0.36–0.63)
	Category 2:	‘Fatty’ II (1.5 to < 2.0)	0.83 (0.69–1.00)
	Category 3:	‘Scattered’ I (2.0 to < 2.2)	1 [Reference]
	Category 4:	‘Scattered’ II (2.2 to < 2.6)	1.43 (1.24–1.64)
	Category 5:	‘Heterogeneous’ I (2.6 to < 2.9)	1.82 (1.59–2.07)
	Category 6:	‘Heterogeneous’ II (2.9 to < 3.2)	2.44 (2.11–2.81)
	Category 7:	‘Extremely dense’ I (3.2 to < 3.6)	2.66 (2.23–3.17)
	Category 8:	‘Extremely dense’ II (≥ 3.6)	3.03 (2.48–3.70)
			ΔLR-*χ*^2^ (*df* = 7) = 375.1

*n* = 132,439

Adjusted hazard ratios from proportional-hazards Cox models for BI-RADS density (time-varying) or the new longitudinal breast density measure (time-varying) as a continuous or eight-category value

Hazard ratios adjusted for age at baseline (continuous) and time-varying BMI (continuous)

‘Time-varying’ means that a woman’s values for breast density and BMI are updated through time, i.e. at each screening examination

Cut points for the eight-category new breast density measure were chosen so that, at baseline mammogram, the proportion of women in the Fatty, Scattered, Heterogeneous and Extremely dense categories are the same as for BI-RADS density, and then split equally into the I and II subgroups within each category

95% CIs from Wald tests

ΔLR-*χ*^2^ represents the additional information in the likelihood-ratio statistic when including breast density

*BI-RADS*  Breast Imaging Reporting and Data System, *BMI* body mass index, *df* degrees of freedom, *95% CI* 95% confidence interval

*Units of longitudinal density are non-standard—interpretation may be facilitated by Fig. 1

In a secondary analysis restricted to women with at least three mammograms (*n* = 76,313 (58% of cohort), 2169 cancers after third mammogram), the gain in statistical information when using the continuous longitudinal density measure was 35% more than when using BI-RADS density (ΔLR-*χ*^2^(2) = 251.2 vs. ΔLR-*χ*^2^(3) = 186.7, Table 3), with respective mean c-indices of 0.63 and 0.62 (mean difference in c-indices = 0.010, 95% CI 0.005–0.015). Using eight-category longitudinal density (ΔLR-*χ*^2^(7) = 246.3), the risk gradient between densest and fattiest breasts was greater for the extremes of the eight-category longitudinal density (HR = 4.8, 95% CI 3.5–6.8) than BI-RADS density (HR = 3.5, 95% CI 2.7–4.5). Using breast density measures at/to the third mammogram and not updating further through time yielded similar results to the main analysis with breast density as a time-varying covariate (Table 3).

**Table 3:** Adjusted hazard ratios for invasive breast cancer by breast density (BI-RADS/longitudinal): subgroup with ≥ 3 mammograms

Model	Density measure		Adjusted hazard ratio (95% CI) (time-varying breast density covariate)	Adjusted hazard ratio (95% CI) (breast density at/to third mammogram)
1	BI-RADS density			
	Fatty		0.52 (0.42–0.65)	0.52 (0.40–0.67)
	Scattered		1 [Reference]	1 [Reference]
	Heterogeneous		1.58 (1.43–1.74)	1.57 (1.42–1.74)
	Extremely dense		1.82 (1.55–2.14)	2.06 (1.77–2.40)
			ΔLR-*χ*^2^ (*df* = 3) = 186.7	ΔLR-*χ*^2^ (*df* = 3) = 175.9
2	Continuous longitudinal density			
	Linear (per unit*)		5.62 (3.25–9.72)	4.99 (2.81–8.84)
	Quadratic (per unit^2^*)		0.82 (0.74–0.90)	0.84 (0.76–0.94)
			ΔLR-*χ*^2^ (*df* = 2) = 251.2	ΔLR-*χ*^2^ (*df* = 2) = 252.0
3	Categorical longitudinal density			
	Category 1:	‘Fatty’ I (< 1.5)	0.47 (0.35–0.63)	0.45 (0.32–0.62)
	Category 2:	‘Fatty’ II (1.5 to < 2.0)	0.73 (0.60–0.89)	0.79 (0.63–0.98)
	Category 3:	‘Scattered’ I (2.0 to < 2.2)	1 [Reference]	1 [Reference]
	Category 4:	‘Scattered’ II (2.2 to < 2.6)	1.24 (1.06–1.45)	1.34 (1.15–1.56)
	Category 5:	‘Heterogeneous’ I (2.6 to < 2.9)	1.58 (1.36–1.84)	1.73 (1.49–2.01)
	Category 6:	‘Heterogeneous’ II (2.9 to < 3.2)	1.98 (1.70–2.32)	2.09 (1.79–2.44)
	Category 7:	‘Extremely dense’ I (3.2 to < 3.6)	2.25 (1.84–2.75)	2.31 (1.89–2.82)
	Category 8:	‘Extremely dense’ II (≥ 3.6)	2.27 (1.81–2.86)	2.92 (2.36–3.62)
			ΔLR-*χ*^2^ (*df* = 7) = 246.3	ΔLR-*χ*^2^ (*df* = 7) = 242.2

*n* = 76,313 (subgroup of women with at least 3 mammograms); follow-up from the third mammogram

Adjusted hazard ratios from proportional-hazards Cox models for BI-RADS density (time-varying or at third mammogram) or the new longitudinal breast density measure (time-varying or to third mammogram) as a continuous or eight-category value

Hazard ratios adjusted for age at baseline (in this instance, where follow-up started at the third mammogram, age at third mammogram was used) (continuous) and BMI (continuous; time-varying or at third mammogram)

‘Time-varying’ means that a woman’s values for breast density and BMI are updated through time, i.e. at each screening examination

Cut points for the eight-category new breast density are the same as those used in Table 2

95% CIs from Wald tests

ΔLR-*χ*^2^ represents the additional information in the likelihood-ratio statistic when including breast density

*BI-RADS*  Breast Imaging Reporting and Data System, *BMI* body mass index, *df* degrees of freedom, *95% CI* 95% confidence interval

*Units of longitudinal density are non-standard—interpretation may be facilitated by Fig. 1

**Fig. 3:**
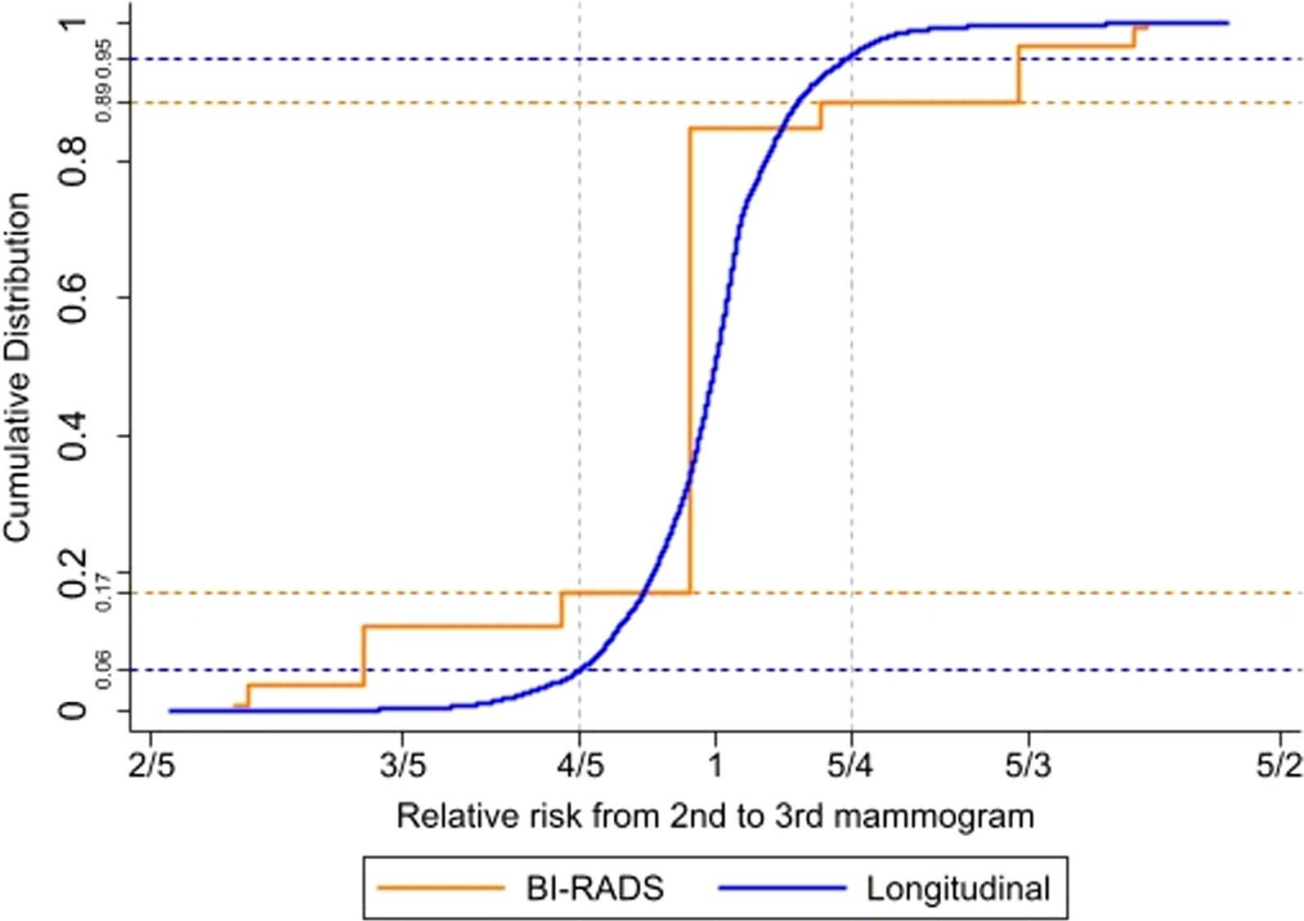
Cumulative distribution functions for relative risk of breast density (BI-RADS/longitudinal) from second to third mammogram. *n* = 76,313 (subgroup of women with at least 3 mammograms). Relative risks were calculated using the hazard ratios for density taken from the proportional-hazards Cox model fitted to the complete cohort for BI-RADS density (time-varying) or the new longitudinal breast density measure (time-varying; continuous; including a quadratic term), age at baseline (continuous) and BMI (time-varying; continuous). Corresponding observed values for BI-RADS density or the new longitudinal breast density measure were from the second and third mammograms in the subgroup of women with at least 3 mammograms. Relative risks from second to third mammogram were normalised so that mean = 1. ‘Time-varying’ means that a woman’s values for breast density and BMI are updated through time, i.e. at each screening examination. Orange line represents the cumulative distribution function for BI-RADS density. Blue line represents the cumulative distribution function for longitudinal density. X-axis on a log scale. *BI-RADS* Breast Imaging Reporting and Data System, *BMI* body mass index

Cumulative distribution plots for relative risk associated with the second and third mammogram suggested that longitudinal density is more stable through time than BI-RADS density: 11% of women had an estimated relative risk outside of the stable range (relative risk was considered to be stable between 4/5 and 5/4) when using longitudinal density, compared with 28% when using BI-RADS density (Fig. 3).

Exploratory analysis including density trajectories showed only a small improvement on the continuous longitudinal density model (ΔLR-*χ*^2^(1) = 4.0, *p* = 0.046), with a 1% gain in statistical information. There was little evidence of an interaction between continuous longitudinal density and age, BMI or race (age at baseline interaction, HR (per 5 yr) = 0.99, 95% CI 0.94–1.03; BMI interaction, HR (per 5 kg/m^2^) = 1.08, 95% CI 0.99–1.17; race interaction, HR (non-White relative to White) = 0.99, 95% CI 0.81–1.21). Longitudinal density performed better than BI-RADS density in both premenopausal (*n* = 87,156, 1581 invasive breast cancers) and postmenopausal (*n* = 45,283, 1123 invasive breast cancers) women (ΔLR-*χ*^2^ was 23% and 21% greater for continuous longitudinal density than BI-RADS density, respectively), and for both oestrogen receptor positive (*n* = 2175) and negative (*n* = 418) subtypes (excluding 111 invasive breast cancers with unknown oestrogen receptor status, *n* = 132,328 in both the oestrogen receptor positive and negative models, ΔLR-*χ*^2^ was 24% and 19% greater for continuous longitudinal density than BI-RADS density, respectively; heterogeneity p-values: linear = 0.816, quadratic = 0.772).

In sensitivity analyses, results were robust to excluding mammograms taken up to 6 months before an incident breast cancer (all women, follow-up from baseline mammogram, ΔLR-*χ*^2^ was 23% greater for continuous longitudinal density than BI-RADS density). We also found similar results when removing mammograms with imputed BMI (*n* = 129,748, 2668 invasive breast cancers, follow-up from updated baseline mammogram, ΔLR-*χ*^2^ was 30% greater for continuous longitudinal density than BI-RADS density). Furthermore, continuous longitudinal density outperformed baseline BI-RADS density (ΔLR-*χ*^2^(2) = 379.6 vs. ΔLR-*χ*^2^(3) = 296.2, respectively, resulting in a ΔLR-*χ*^2^ that was 28% greater for continuous longitudinal density than baseline BI-RADS density; baseline BI-RADS density was adjusted for age at baseline and baseline BMI). This suggests that a greater gain in aetiological information can be obtained by including multiple historical measures of mammographic density instead of a single measure that reflects a woman’s ‘peak’ available breast density. Four-category longitudinal breast density also performed slightly better than BI-RADS density (ΔLR-*χ*^2^(3) = 312.3 vs. ΔLR-*χ*^2^(3) = 307.7), indicating that the improvement in statistical ability when using longitudinal density is explained by more than just longitudinal density being measured continuously compared with categorical BI-RADS. Finally, in a post hoc sensitivity analysis, we tested whether the superior performance of longitudinal breast density compared with BI-RADS density could be due to the incorporation of an interaction term between age and BMI in the calculation of the longitudinal breast density measure. However, a proportional-hazards model including a single (time-varying) BI-RADS density measure, age at baseline, BMI (time-varying) and an age–BMI interaction did not perform as well as the proportional-hazards model including longitudinal density (time-varying; including a quadratic term), age at baseline and time-varying BMI (ΔLR-*χ*^2^(4) = 308.8 vs. ΔLR-*χ*^2^(2) = 379.6).

## Discussion

Using a woman’s longitudinal history of breast density could improve breast cancer risk estimation in women attending mammography screening over using a single measurement alone. Our proposed longitudinal breast density measure provided a gain in statistical information that was 23% more than that provided by BI-RADS density. Women in the highest longitudinal density category had a sixfold greater risk of developing breast cancer than women in the lowest category, compared with a fourfold difference when using BI-RADS density. This greater spread of risk with eight-category longitudinal density (which demonstrates the greater spread of risk that is achievable when using the continuous longitudinal density measure) suggests a potential for improved risk stratification with the continuous longitudinal density measure. We consider there to be two main benefits of using repeated mammograms and deriving a longitudinal breast density measure. Firstly, the longitudinal breast density measure is likely to be reflecting a woman’s long-term average exposure to breast density and therefore aligning with Boyd et al.’s model of breast tissue ageing which suggests that cumulative rate of exposure to hormones, and consequently breast density, is positively associated with breast cancer risk [27, 28]. Secondly, since the longitudinal breast density measure appears to be more stable over time than a single measure of BI-RADS density, it is likely that it reduces variation in estimates of an individual’s visual assessment of breast density and therefore also breast cancer risk between screens. This should prevent fluctuations in the classification of women into different risk categories through time. We also note that the improvement in discrimination when using longitudinal breast density was relatively small, suggesting that the benefit of the measure is more so in its ability to improve the reliability and accuracy of a woman’s breast density measurements and personalised estimate of breast cancer risk than in its ability to differentiate between women who will, and will not, go on to develop breast cancer. However, given that the longitudinal density measure is likely to reduce the amount of noise in data, its utilisation should result in an improvement in the performance of established breast cancer risk models that currently use BI-RADS density.

These results support previous findings that suggest an improvement in predictive ability of breast cancer risk estimation when using density values from more than one time point [6]. Kerlikowske et al. assessed BI-RADS density in a screening cohort of ~ 700,000 women from the BCSC, where a two-measure density score was developed combining first and last BI-RADS density measures taken on average 1.8 years apart [6]. They found an improvement in the BCSC 5-year risk model when using their two-measure score compared with a one-measure score, whereby the area under the receiver operating curve (AUC) increased by ~ 0.005. Our study (which includes some of the women in Kerlikowske et al.’s study) also found a slight improvement in discriminatory accuracy when using multiple density measures (compared with a single density measure, the concordance index increased by ~ 0.008). Several other studies have also made use of more than one serial density value [4, 5], with most suggesting an association between change in density and breast cancer risk [5, 31–41], demonstrating the benefit of including more than one time point for breast cancer risk estimation. However, these association studies did not focus on evaluating the predictive ability of using serial density measurements to estimate future risk of breast cancer.

There is a question of whether the longitudinal breast density measure improves accuracy or whether it provides aetiological information. In our view, the main statistical advantage of longitudinal density is to reduce measurement error and improve reliability and accuracy by functioning as a shrinkage estimator making use of multiple data points. This was apparent from our results because the predictive ability of longitudinal density improved by a greater amount when it was included in the proportional-hazards survival analysis as a continuous variable than as a categorical variable. This result also highlights the advantage of using finer-grained descriptions of a woman’s history of breast density. In addition, we initially hypothesised that including information on the rate of change of each woman’s density might be informative (analysis including random slopes in the model). However, our data suggested only a small effect on predictive ability when including each woman’s individual slope. Whilst this finding indicates a high level of density tracking (which is consistent with earlier work [42, 43]), it differs from the results of a recent study which reported a better model fit when using both current mammographic density and individual slope compared with current mammographic density alone [44]. A possible explanation for the contrasting findings with our study is that different density measures were used. [BI-RADS density was used in our study, and STRATUS (an automated area-based measure of absolute density) was used in Illipse et al.] It would be worthwhile investigating the longitudinal breast density method using different measures of mammographic density such as the aforementioned. Another consideration of the longitudinal breast density measure is that, whilst the method is clearly most useful when a woman has more than one density measure, it may also improve reliability of breast density measurements even with just a single density score. This is because it borrows strength from other women of the same age and body mass index, and shrinks the estimates accordingly. For example, one might expect a 40-yr-old woman with healthy weight and BI-RADS density D to have truly denser breasts than a 70-yr-old woman with obesity and BI-RADS density D; and this would be reflected in the proposed algorithm.

The major strength of this study is the use of a large cohort with repeated measures of breast density through time, and the development and predictive evaluation of a new method to assess serial density, particularly using more than two measures arbitrarily spaced through time. This might make longitudinal density a particularly useful tool for clinical practice where women can have several mammograms taken at any point in time. We additionally observed improvements when assessing women with at least three prior mammograms, which demonstrates an increasing value of the method with more data. Therefore, the benefit of using longitudinal density to assess breast cancer risk in terms of reducing measurement error and preventing fluctuations in the classification of women into different risk categories through time may be improved further by updating the measure at each screening examination to provide more density values. Another advantage of using longitudinal breast density is that it would help to identify women with persistently dense breasts who may be eligible for supplemental screening. This is an important piece of information that cannot be discerned from a single BI-RADS density measure. In addition, the beneficial consequence of having greater stability when using longitudinal breast density compared with BI-RADS density is likely to have implications for breast cancer screening programmes which employ a risk-based approach. For instance, according to the UK’s National Institute for Health and Care Excellence guidelines and previous studies assessing the application of these guidelines to population risk assessment, breast cancer risk thresholds of ≥ 8% and 5–8% absolute 10-year risk may be used to classify women into high- and moderate-risk groups, respectively [3, 45, 46]. Therefore, in the UK context, if a group of high-risk women with an 8–10% absolute 10-year risk of breast cancer (based on an established risk model) were to experience a 20% reduction in relative risk (i.e. relative risk = 4/5), then 17% of these women would be reclassified as moderate risk if using BI-RADS density, compared with only 6% if using longitudinal breast density (under the assumption that the distribution in Fig. 3 holds for comprehensive risk assessment within this group). Such a large difference in reclassification would have important ramifications for women who are truly at a high risk of breast cancer and may benefit from enhanced screening, but would be ineligible for it due to omission of information from previous mammograms. Furthermore, in view of the recent U.S. Food and Drug Administration regulation updates requiring patient mammography reports to indicate whether they have ‘dense’ or ‘non-dense’ breast tissue [47], and given that longitudinal density is more stable over time than a single density measure, the use of the longitudinal density measure could help to mitigate confusion amongst patients if their density values were to vary between screening visits. (This is especially relevant for women whose BI-RADS density oscillates between B and C.)

Limitations of the study include the statistical models used. In particular, BI-RADS density categories were modelled as quantitative integer values to crudely approximate a linear association between density and age and BMI in the linear mixed model. There is some justification for this because the relative hazard associated with BI-RADS density adjusted for age and BMI is approximately 1, 2, 3, 4 (cf. comparing relative differences in Table 2). However, alternative models, such as joint models, might better fit the data and improve predictive ability [48]. Furthermore, the linear mixed model used to develop longitudinal density was adjusted for age and BMI only. Adjusting longitudinal density for additional confounders such as hormone therapy use, benign breast disease or reproductive factors might improve its approximation; although we note that these adjusting factors may be more relevant when predicting breast cancer risk than breast density. Moreover, in a recent association study examining the relationship between change in volumetric percentage density and subsequent breast cancer risk, each breast was considered independently in the statistical model [49]. It is possible that modelling each breast independently could provide additional aetiological information. This approach was not possible in our analysis, however, because the density values at each time point were measured as a combined assessment of the four breast view combinations available to the interpreting radiologist at the screening examination. Another limitation is that the algorithm for calculating longitudinal density is not straightforward to implement using a simple rule. However, it is less complicated to implement in computer systems than fully automated measures of breast density, and the algorithm to evaluate this new measure is fully available in Supplementary Methods, Additional file 1. Finally, since the focus of this study was on evaluating the predictive performance of longitudinal breast density above that of age and BMI, rather than in the context of established risk models, we did not adjust for additional breast cancer risk factors. Future work will aim to validate the longitudinal breast density method in independent datasets, as well as assess the value of incorporating the measure into established breast cancer risk models such as the IBIS/Tyrer–Cuzick algorithm [50]. Ultimately, by adapting such risk models to include a woman’s historical information, dynamic risk prediction could be made possible. Patient data could be updated regularly, and accurate and reliable estimates of breast cancer risk (that take into account all of the woman’s longitudinal information) could be provided at each new screening visit.

## Conclusions

We conclude that estimating mammographic density using a woman’s history of breast density is likely to be more reliable than using the most recent observation only, which may lead to more reliable and accurate estimates of individual breast cancer risk. Longitudinal breast density has the potential to improve personal breast cancer risk estimation in women attending mammography screening.

### Supplementary Information

**Additional file 1.** Title of data: Supplementary Figure S1; Supplementary Methods; Supplementary Table S1. Description of data: flow chart of mammograms and women included in the analysis; calculation of the at-risk concordance index, an intuitive explanation of the longitudinal density measure, the algorithm to calculate the new longitudinal breast density measure; linear mixed model fit for the new continuous longitudinal breast density measure.

## Data Availability

The datasets analysed during the current study are not publicly available because they contain potentially identifiable information (e.g. dates of diagnoses and treatment) that cannot be shared openly without human subjects’ approval and data use agreements, but are available from Kaiser Permanente Washington Health Research Institute on reasonable request, subject to data sharing agreements. R files containing the algorithm and model parameters to calculate the new longitudinal breast density measure are fully available at https://www.github.com/emmaatakpa/longitudinal-breast-density.

## Abbreviations

AUC: Area under the receiver operating curve
BCSC: Breast Cancer Surveillance Consortium
BI-RADS: Breast Imaging Reporting and Data System
BMI: Body mass index
DCIS: Ductal carcinoma in situ
df: Degrees of freedom
HR: Hazard ratio
IQR: Interquartile range
95% CI: 95% Confidence interval
ΔLR-*χ*^2^: Likelihood-ratio statistic
