# Supplementary material for: Development and evaluation of a method to assess breast cancer risk using a longitudinal history of mammographic density: a cohort study
Source: Breast Cancer Res. 2023 Nov 24;25:147. doi: 10.1186/s13058-023-01744-y (PMC10668455; doi:10.1186/s13058-023-01744-y)
Supplement: Supplementary file 1 — Additional file 1. Title of data: Supplementary Figure S1; Supplementary Methods; Supplementary Table S1. Description of data: flow chart of mammograms and women included in the analysis; calculation of the at-risk concordance index, an intuitive explanation of the longitudinal density measure, the algorithm to calculate the new longitudinal breast density measure; linear mixed model fit for the new continuous longitudinal breast density measure. [file 13058_2023_1744_MOESM1_ESM.docx]

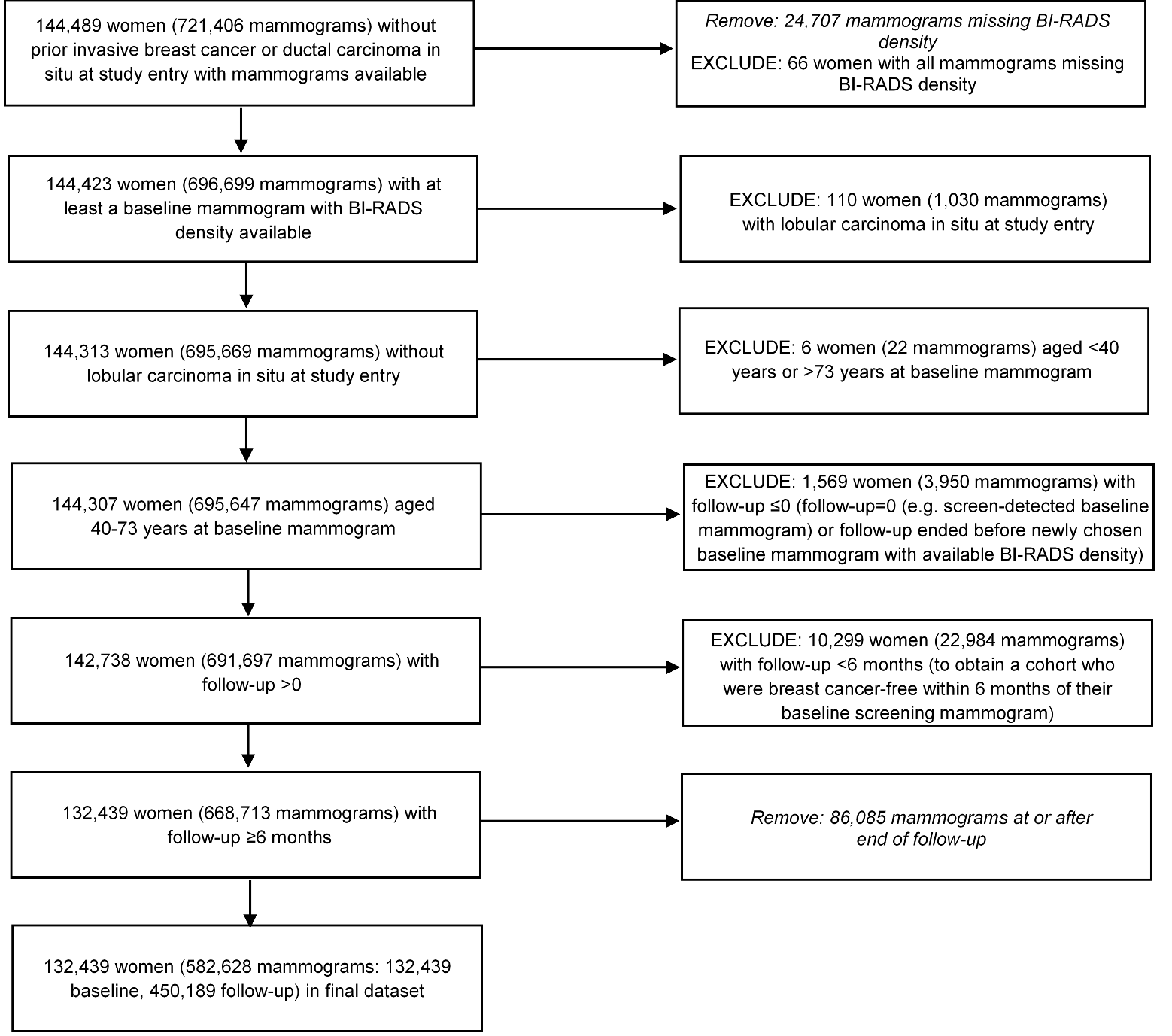


Supplementary Figure S1: Flow chart of mammograms and women included in the analysis. BI-RADS = Breast Imaging Reporting and Data System.

Supplementary Methods

Calculation of the at-risk concordance index

Survival status for woman $i=1,\ldots,n$is denoted $\left( t_{i},\delta_{i} \right)$ for the time $t_{i}$ of breast cancer event $\left( \delta_{i}=1 \right)$ or censoring $\left( \delta_{i}=0 \right).$ A risk score, $r_{ij}$, is determined for each woman $i=1,\ldots,n$at each breast cancer event $j=1,\ldots,m$using the estimated hazard ratio from the proportional-hazards Cox model. That is, $r=exp\left( \boldsymbol{\beta x} \right)$, where $\boldsymbol{x}\mathbf{=}(x_{1},x_{2},x_{3})$ includes age $(x_{1})$, body mass index (BMI)${(x}_{2})$ and breast density${(x}_{3})$, with corresponding parameters $\boldsymbol{\beta}^{T}\mathbf{=}(\beta_{1},\beta_{2},\beta_{3})$. BMI and breast density values are updated through time, hence $x_{2}$ and $x_{3}$ are time-varying covariates.

At each breast cancer event $j=1,\ldots,m$ (occurring at time, $S_{j}$), a concordance index, $C_{j}$, is defined as:

$$C_{j}=\frac{\sum_{\begin{aligned} i=1 \\ i\neq\omega_{j} \end{aligned}}^{n} I\left( t_{i}\geq S_{j} \right)\left\{ I\left( r_{ij}<\tilde{r}_{j} \right)+\frac{1}{2}I\left( r_{ij}=\tilde{r}_{j} \right) \right\}}{\sum_{\begin{aligned} i=1 \\ i\neq\omega_{j} \end{aligned}}^{n} I\left( t_{i}\geq S_{j} \right)} ; (*)$$

where the risk score of the woman with the event ${j=\tilde{r}}_{j}$, and the index of the woman with the event $j=\omega_{j}$. That is, $C_{j}$ is the proportion of women with a risk score less than or equal to the risk score of the woman generating the breast cancer event (out of the total number of women still at-risk at the time of the breast cancer event).

Generalising to include ties ($\geq$1 woman ($=\tilde{n}_{j}$) with an event at the same time, $S_{j}$), $(*)$ is calculated separately for each tied woman $k=1,\ldots, \tilde{n}_{j}$ at event $j$. The index, $\omega_{j}$, is extended to be a vector of indices, $\boldsymbol{\omega}_{\boldsymbol{j}}=(\omega_{j1},\ldots,\omega_{j\tilde{n}_{j}})$, and the risk score of the tied woman $k$ at event $j{=\tilde{r}}_{jk}$. Hence:

$$C_{jk}=\frac{\sum_{\begin{aligned} i=1 \\ i\notin\boldsymbol{\omega}_{\mathbf{j}} \end{aligned}}^{n} I\left( t_{i}\geq S_{j} \right)\left\{ I\left( r_{\mathrm{ij}}<\tilde{r}_{\mathrm{jk}} \right)+\frac{1}{2}I\left( r_{\mathrm{ij}}=\tilde{r}_{\mathrm{jk}} \right) \right\}}{\sum_{\begin{aligned} i=1 \\ i\notin\boldsymbol{\omega}_{\mathbf{j}} \end{aligned}}^{n} I\left( t_{i}\geq S_{j} \right)}$$

A yearly mean concordance index, $yC_{z}$, is presented at each yearly interval $z=1,\ldots, 18$, starting at 0.5 years. So, for example, the yearly mean concordance index between 0.5 years and 1.5 years is defined as:

$$yC_{1}=\frac{\sum_{j=1}^{m} \sum_{k=1}^{\tilde{n}_{j}} C_{\mathrm{jk}} \left\{ I\left( 0.5\leq S_{j}<1.5 \right) \right\}}{\sum_{j=1}^{m} \tilde{n}_{j} \left\{ I\left( 0.5\leq S_{j}<1.5 \right) \right\}}$$

The maximum follow-up time is 19 years, hence the final $yC_{z} (=yC_{18})$ is calculated between 17.5 years and 18.5 years and the 4 women who developed breast cancer $\geq$18.5 years are excluded.

The standard error on each $yC_{z}$ is calculated by estimating the variance about the mean, $yC_{z}$, (variance generated by the women with a breast cancer event, $j$, occurring in yearly interval, $z$).

So, for example, for $z=1$ (between 0.5 years and 1.5 years), the variance about the mean, $yC_{1}$, is defined as:

$$y_{1}=\frac{\sum_{j=1}^{m} \sum_{k=1}^{\tilde{n}_{j}} \left( C_{\mathrm{jk}}-yC_{1} \right)^{2} \left\{ I\left( 0.5\leq S_{j}<1.5 \right) \right\}}{\sum_{j=1}^{m} \tilde{n}_{j} \left\{ I\left( 0.5\leq S_{j}<1.5 \right) \right\}}$$

Hence, the standard error (SE) on $yC_{1}$ is calculated as:

$$\mathrm{SE}_{1}=\sqrt{\frac{y_{1}}{\sum_{j=1}^{m} \tilde{n}_{j} \left\{ I\left( 0.5\leq S_{j}<1.5 \right) \right\}}}$$

An intuitive explanation of the longitudinal density measure

Using two time points, there are 4 x 4 = 16 possible BI-RADS density combinations (first then last (i.e., most recent) mammogram: AA, AB, AC, AD, BA, BB, BC, etc.).

If you were categorising them into 16 groups, you might have (in this example, the most recent time point is given priority; so, for CD and DC, CD is denser than DC etc.):

16 = DD = densest category

15 = CD

14 = DC

...

1 = AA = fattiest category

If you were categorising them into 8 groups, you might have (in this example, the most recent time point is given priority; so, for CD and DC, CD is denser than DC etc.):

8 = DD, CD = densest category

7 = DC, CC

…

1 = BA, AA = fattiest category

The linear mixed model essentially uses a similar approach except, instead of categories, it uses numbers, where D=4, C=3, B=2, A=1.

So, DD = 4, CD=3.5, DC=3.5, CC=3, etc.

However, the linear mixed model also adjusts for age (and thereby also timing of the mammogram) and body mass index. Therefore, the new longitudinal breast density measure is a continuous variable that can be updated at each new screen using information from the previous mammograms.

The algorithm to calculate the new longitudinal breast density measure

R files containing the algorithm and model parameters to calculate the new longitudinal breast density measure are fully-available at <https://www.github.com/emmaatakpa/longitudinal-breast-density>.

Supplementary Table S1: Linear mixed model fit for the new continuous longitudinal breast density measure

| Fixed effects | | | | |
| --- | --- | --- | --- | --- |
| Variable | β-coefficient | | Robust standard error* | |
| Intercept | 2.9659 | | 0.0044 | |
| Age (per 5 years) | 0.0491 | | 0.0079 | |
| Age^2^ (per 5^2^ years) | -0.0780 | | 0.0048 | |
| Age^3^ (per 5^3^ years) | 0.0127 | | 0.0011 | |
| Age^4^ (per 5^4^ years) | -0.0006 | | 0.0001 | |
| BMI (per kg/m^2^) | -0.0584 | | 0.0005 | |
| Age x BMI (per 5 years; per kg/m^2^) | 0.0033 | | 0.0001 | |
| Random effects | | | | |
| Variable | Standard deviation | Robust standard error* | Correlation | Robust standard error* |
| Intercept | 0.5865 | 0.0027 | -0.4590 | 0.0068 |
| Age (per 5 years) | 0.1107 | 0.0010 |  |  |

BI-RADS density (integer) fitted against age (continuous) and BMI (continuous).

Age from 40 years, BMI from 25kg/m^2^.

BI-RADS = Breast Imaging Reporting and Data System; BMI = Body mass index.

*Standard errors calculated using a robust sandwich estimator.
